# Modeling Airflow and Particle Deposition in a Human Acinar Region

**DOI:** 10.1155/2019/5952941

**Published:** 2019-01-14

**Authors:** Arun V. Kolanjiyil, Clement Kleinstreuer

**Affiliations:** ^1^Department of Mechanical & Aerospace Engineering, North Carolina State University, Raleigh, NC 27695, USA; ^2^Joint UNC-NCSU Department of Biomedical Engineering, North Carolina State University, Raleigh, NC 27695, USA

## Abstract

The alveolar region, encompassing millions of alveoli, is the most vital part of the lung. However, airflow behavior and particle deposition in that region are not fully understood because of the complex geometrical structure and intricate wall movement. Although recent investigations using 3D computer simulations have provided some valuable information, a realistic analysis of the air-particle dynamics in the acinar region is still lacking. So, to gain better physical insight, a physiologically inspired *whole acinar model* has been developed. Specifically, air sacs (i.e., alveoli) were attached as partial spheroids to the bifurcating airway ducts, while breathing-related wall deformation was included to simulate actual alveolar expansion and contraction. Current model predictions confirm previous notions that the location of the alveoli greatly influences the alveolar flow pattern, with recirculating flow dominant in the proximal lung region. In the midalveolar lung generations, the intensity of the recirculating flow inside alveoli decreases while radial flow increases. In the distal alveolar region, the flow pattern is completely radial. The micron/submicron particle simulation results, employing the Euler–Lagrange modeling approach, indicate that deposition depends on the inhalation conditions and particle size. Specifically, the particle deposition rate in the alveolar region increases with higher inhalation tidal volume and particle diameter. Compared to previous acinar models, the present system takes into account the entire acinar region, including both partially alveolated respiratory bronchioles as well the fully alveolated distal airways and alveolar sacs. In addition, the alveolar expansion and contraction have been calculated based on physiological breathing conditions which make it easy to compare and validate model results with in vivo lung deposition measurements. Thus, the current work can be readily incorporated into human whole-lung airway models to simulate/predict the flow dynamics of toxic or therapeutic aerosols.

## 1. Introduction

The alveolar region encompasses millions of alveoli specifically designed to allow efficient gas diffusion during the normal breathing process [1]. Flow behavior and particle deposition in the alveolar region are not yet fully understood because of the complex geometrical structure [2, 3]. Understanding the flow and particle behavior in the alveolar region is important because inhaled aerosols depositing in the alveoli can lead to severe lung diseases [4–6]. In case of therapeutic drug targeting [7, 8], the prediction of airflow and particle deposition in the alveolar region is necessary for the treatment of pulmonary diseases as well as drug-aerosol transfer to systemic regions.

Although the acinar region is the most vital part of the lung, only limited information is available regarding its geometry because of its inaccessibility. Current limitations in obtaining detailed images of these micron structures also restrict detailed fluid-particle dynamics studies [5, 9]. Similar to the conducting region, the acinar region is composed of repeating bifurcating alveolar ducts with alveoli arranged around the conduits [10, 11]. Each alveolus may be approximated as a spherically shaped cup [12], with one opening to the airway, tightly packed around the alveolar ducts in order to optimize the alveolar surface area [11]. These alveoli are separated by a thin tissue layer called septa which contain a multitude of blood vessels [10]. Several studies have used lung casts to analyze the geometrical structure of the acinus, and the reported alveoli topology varies from study to study [1, 10–14]. Recently, micro X-ray computed tomography (micro-CT) has been used to study morphology and geometric structure of rodent acinar lung airways [14, 15]. Three-dimensional (3D) reconstructions of acinar airways from high-resolution CT images show the irregular nature of the alveolar topology but closely resemble the spheroidal topology [13]. Polygonal space-filling structure of the alveoli has also been suggested in several studies [4, 16–19]. However, with these sac shapes, it is difficult to assess the bifurcation characteristics of the alveolar ducts, and it may not be able to replicate the partially alveolated transitional respiratory bronchioles. Furthermore, recent studies indicate that the topological features of alveoli may not be significant in distal airways and sacs [17, 20].

Traditionally it has been thought that the low Reynolds number flow in the alveolar region is completely reversible. However, this assumption was reexamined after the bolus inhalation experiments by Heyder et al. [21] in which they showed that irreversible convective mixing exists in the peripheral lung airways. This may lead to high deposition of inhaled particles deep inside the lung. In spite of the evidence showing convective mixing of acinar airflow, these in vivo experiments could not provide detailed information regarding trajectories and local deposition of particles in submillimeter alveolar structures. As an alternative, numerical analyses were used, based on simplified alveolar configurations, still highlighting the prominent flow characteristics in the alveolar region, including recirculation and chaotic mixing [22]. These earlier findings were again confirmed based on additional experimental and numerical results [5, 13, 23, 24]. Nevertheless, it is still not clear how the alveolar structure at the microlevel changes during breathing [25]. Several explanations have been put forward to explain the mechanism; yet, no consensus exists [26]. The common hypotheses are the following: (1) isotropic balloon-like expansion of alveoli; (2) expansion followed by shape change; (3) recruitment and derecruitment of alveoli; and (4) anisotropic accordion-like shape changes [27, 28]. Studies have pointed out that the deforming alveoli primarily contribute to changes in lung volume, while the deformations of airways and ducts have a considerably lower influence [29].

In addition to the airflow, several studies have indicated that the particle transport and deposition in the alveolar airways are significantly affected by the alveolar-wall motion [30, 31], predominantly affecting submicron particles [32]. In these investigations, it was noticed that the particle deposition pattern in the alveoli was mostly heterogeneous; however, the alveolar entrance ring was an important site for particle deposition [32, 33]. Similarly, heterogeneous deposition of particles on the duct and alveolar walls was observed in multigenerational, bifurcating alveolar models [34, 35]. However, concentrated particle deposition was noticed at the bifurcation ridge, indicating the significance of alveolar airway bifurcation [36–38]. Recently, 3D space-filling alveolar models have been used in alveolar airway simulations [4, 5, 17, 20, 39–41]. The alveolar airways were developed using repeated polyhedral units assuming equal space-filling alveoli and the interconnecting faces were removed to form the alveolar ducts. Most of these simulations were conducted assuming uniform ventilation in all alveoli; however, anisotropic ventilation due to heterogeneous alveolar-wall expansion may also contribute to the chaotic nature of the alveolar flow [39]. Even though the space-filling polyhedral models can accommodate multiple alveolated ducts, these models cannot account for the variation in airway bifurcations. Additionally, these models have not been shown to predict whole acinar lung deposition; hence, they have not been incorporated to study the deposition in a whole-lung model, with the exception of Khajeh-Hosseini-Dalasm and Longest [41]. More recently, microfluidic lab-on-chip models of the acinar generations have been developed to study the rhythmic motion of the alveoli as well as its impact on alveolar flow pattern and particle transport [42, 43]. Initial results have shown streamline crossing of particles due to the dominant effects of gravity and diffusion, leading to site-specific deposition depending on particle size.

The objective of the present study was to develop a *full* human acinar airway model to simulate and analyze micron/submicron particle transport/deposition in these lower lung airways. The current acinar model simulates the inhalation-exhalation process, i.e., the expanding and contracting motions of the alveoli. Hence, it can replicate all physiological breathing modes. Furthermore, the current comprehensive model takes into account the variation of alveolar tissue dynamics for each airway generation. Of special interest is the interplay between alveolar-wall deformation and particle deposition due to gravity and secondary flow. As mentioned, the comprehensive acinar model can be readily incorporated into human whole-lung airway models to simulate/predict the fate of inhaled toxic or therapeutic aerosols.

## 2. Methods

### 2.1. Model Geometry

**Figure 1:**
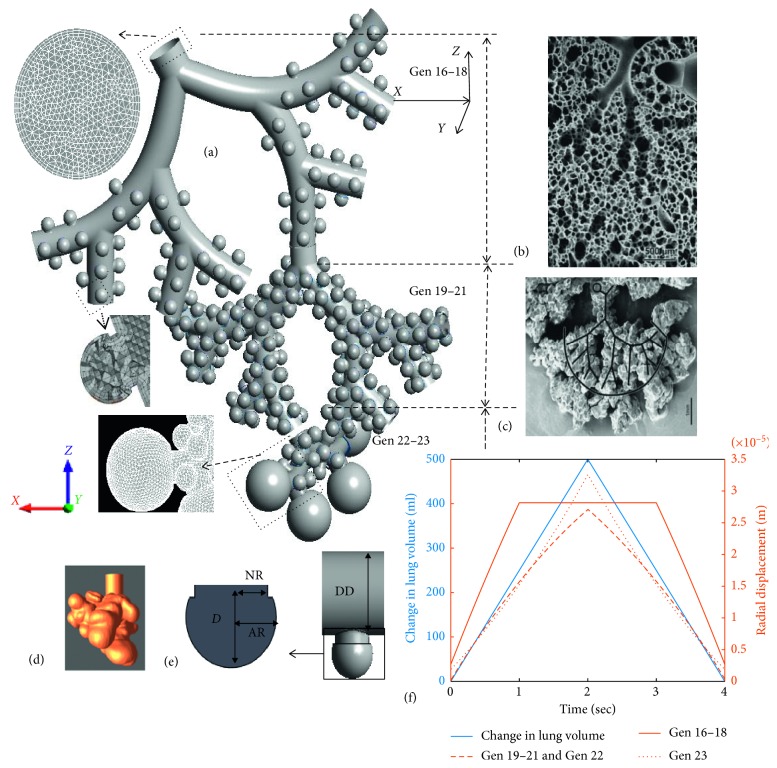
(a) Final assembled full alveolar model; (b) image of terminal conducting airways ending in an acinar region from a lung cast (source [45]); (c) image of fully alveolated airways from a lung cast (source [45]); (d) image of a lung airway sac (source [46] republished with permission of ASME and permission conveyed through Copyright Clearance Center, Inc.); (e) representative alveoli attached duct; and (f) change in lung volume for a 15 LPM breathing flow rate with inhalation period of 2 s, tidal volume of 500 ml, and the corresponding radial displacement vectors for the alveoli in each lung generations.

For the comprehensive acinar model, spherical alveoli were attached to triple bifurcation units (TBUs), where the alveoli were separated by a minimum distance to allow for expansion, representing the septa that separate the alveoli (Figure 1). The spherical shape of the alveoli was selected based on observations by Hansen and Ampaya [12] and Harding and Robinson [13]. A cylindrical projection from the lumen was used to anchor the spherical alveolus to the duct. The final alveolus has a 3/4 spheroid shape. The partial spheroid with the neck was arbitrarily assembled on the surface of the duct so that the alveolar neck opens to the duct. A single alveolus can be described using four parameters: alveolus radius (AR), neck radius (NR), alveolus depth (*D*), and the duct diameter (DD) (Figure 1(e)). The geometrical assumptions have been verified by the morphometric study using human lung casts and images (Figures 1(b)–1(e)) ([13]). In general, the initial dimensions (at time *t* = 0 or at functional residual capacity (FRC)) of the alveoli are given in Table 1 [44]. Depending on the inhalation condition, the alveolus radius and alveolus depth may change with time; however, alveolar neck and airway duct were assumed to be rigid. This assumption is based on previous investigations which pointed out that at shallow breathing conditions, the deforming alveoli primarily contribute to changes in lung volume, while the deformation of airways and ducts are considerably less when compared to the alveoli [29]. The duct diameter for each model was based on the scaled Weibel's symmetrical lung model. The size of the alveoli has not been varied with generation; however, variation in duct diameter with lung generation number was considered. Additionally, the deformation scale of each alveolus depends on the generation number of the airway, which resulted in variation in alveolar size with respect to the airway generation. These alveolar designs were further modified in terms of triple bifurcation units (TBUs) in order to represent alveolar airway generations from 16 to 18 and 19 to 21. A double bifurcation unit (DBU) was used to represent the airway generations 22 and 23. This design of the acinar airways follows a symmetric dichotomous branching structure as reported in many lung-cast studies [10, 11, 45, 46]. The first TBU represents a partially alveolated respiratory or transitional airways. Details regarding the duct dimensions and the number of alveoli per generation are given in [44]. Instead of using separate alveoli, a quasispherical alveolus (closed sac) was used to represent generation 23. The total volume of the 23^rd^ generation was conserved in the model by creating a partial spheroid with the same total volume as in generation 23 (i.e., duct with 17 alveoli). As reported previously, in the terminal alveolar airways, the demarcation of alveoli is not obvious, i.e., they rather form clusters, which results in reduced surface areas. Additionally, recent studies have reported larger sizes for terminal alveoli [47]. The final assembled human acinus and images of human lung casts are shown in Figure 1. Following previous analyses, only one TBU per generation was modeled and the TBU for generations 19–21 and DBU for 22-23 were fitted to one of the outlets of the previous generation [48–50].

### 2.2. Wall Motion and Boundary Conditions

The wall motion of the alveoli surface was incorporated so that the breathing mechanism due to the negative alveolar pressure can be reproduced. The alveoli were assumed to deform isotropically, i.e., balloon-like with preserving shape, while the deformation rates determine the inhalation and exhalation flow rates [26, 27, 29]. The increase or decrease in volume of the alveoli produces a differential pressure which pulls in or expels air, allowing for the simulation of any realistic waveform. Depending on the flow rates, a deformation vector for the alveoli was estimated to produce the required volume changes. For example, the increase in volume from the initial functional residual capacity (FRC) can be represented using the following equations:

(1) $$\begin{array}{c} V_{\text{FRC}}=f\frac{4}{3}\pi r^{3}, \end{array}$$

(2) $$\begin{array}{c} V_{\text{FRC}}+dV=f\frac{4}{3}\pi {\left(r+dr\right)}^{3}=f\frac{4}{3}\pi \left(r^{3}+dr^{3}+3r\;\;dr\left(r+dr\right)\right), \end{array}$$

(3) $$\begin{array}{c} dV=f\frac{4}{3}\pi \left(dr^{3}+3r\;\;dr\left(r+dr\right)\right), \end{array}$$

where *V*_FRC_ is the alveolus volume at FRC, *f* is the fractional volume of the spherical cap representing an alveolus (*f* = 0.75) and the alveolar sac (*f* = 0.97), and *dV* is the differential increase in volume due to the radial expansion *dr*.

Assuming a symmetric dichotomous branching structure of the lung, the inhalation flow rate through any generation will be the flow rate measured at the oral inlet divided by the number of airways in that generation. This can be represented as follows:

(4) $$\begin{array}{c} {\dot{M}}_{g}=\frac{{\dot{M}}_{\text{inlet}}}{2^{g}},\\ {\dot{V}}_{g}=\frac{{\dot{V}}_{\text{inlet}}}{2^{g}}, \end{array}$$

where ${\dot{M}}_{\text{inlet}}$ is the mass flow rate at the oral inlet, ${\dot{M}}_{g}$ is the mass flow rate through the airway generation *g*, ${\dot{V}}_{\text{inlet}}$ is the volume flow rate at oral inlet, and ${\dot{V}}_{g}$ is the volume flow rate through the airway generation *g*.

As mentioned, the airflow rate is generated via the alveolar deformation and the deformation rate varies with the local TBU. The total number of alveoli is 84, 182, 25, 4 in TBU 16–18, 19–21, 22, and 23, respectively, and their corresponding alveolar volume fraction per TBU is 0.03, 0.25, 0.27, and 0.45. The alveoli in each TBU deform in order to produce a fraction of the oral inhalation flow rate which is equal to the total flow rate times the alveolar volume fraction in the TBU. This can be mathematically represented using

(5) $$\begin{array}{c} {\dot{V}}_{\text{TBU}}\left(t\right)=V_{\text{TBU}}^{\text{FRC}}+{\dot{V}}_{\text{inlet}}\frac{V_{\text{TBU}}^{\text{FRC}}}{\sum_{g=17}^{23}V_{g}^{\text{FRC}}}, \end{array}$$

where ${\dot{V}}_{\text{TBU}}\left(t\right)$ is the instantaneous volume of the TBU at any time *t*, *V*_TBU_^FRC^ is the volume of the TBU at FRC, and *V*_*g*_^FRC^ is the volume of the airway generation *g*. The alveolar volume fraction within a TBU is represented using the ratio *V*_TBU_^FRC^/∑_*g*=17_^23^ *V*_*g*_^FRC^.

Additionally, the individual alveolus wall deformation can be estimated by dividing the fractional volume flow rate per TBU with the number of alveoli in that TBU. The instantaneous flow rate *dV*/*dt* in a particular lung generation for any given inhalation condition can be predetermined, and hence the corresponding alveolar deformation can be estimated by solving equation (3) for *dr*/*dt*. Figure 1(f) shows the change in lung volume with respect to a breathing cycle as well as the corresponding radial displacement vectors for the alveoli in each lung generations. The breathing conditions included a flow rate of 15 LPM, inhalation period of 2 s, and a corresponding tidal volume (TV) of 500 ml. The contraction of the alveoli was modeled by following a reverse path from the expanded volume to the original FRC volume at an identical deformation rate, assuming isotropic volume expansion/contraction, i.e., with negligible geometrical hysteresis.

As suggested in previous studies, the rhythmic expansion and contraction of the lung is assumed to be geometrically self-similar in nature [19]. The presence of surfactant also helps the alveoli to expand and contract at the same rate. The control of liquid surface tension by the surfactants and nonlinear elasticity of the lung tissue prohibits expansion of alveoli beyond a specific radius [51]. In order to replicate the physiological breathing motion of the alveoli, the deformation of the alveoli was restricted after a specified increase in alveolar radius. This final radius was estimated based on the self-similarity assumption. For an average human adult with FRC of 3000 ml and total lung capacity of 6000 ml, the maximum increase in alveolar radius is estimated to be 0.26 times the initial radius (Figure 1(f)). Measured alveolar distention values are in close range with the assumed values [26]. It is quite difficult to measure the exact stress-strain relationship in the alveoli during different levels of inflation. The nonlinear alveolar tissue dynamics and the presence of liquid lining layer and surfactants inside alveoli may limit the stretching of the alveolar walls after an initial expansion [52]. So, in the present study, this effect was simulated by assuming that after 500 ml of tidal volume, the expansion rate was reduced to half due to strain-stiffening of the biological tissue. Once the alveoli reached their maximum capacity of deformation, the rates of the alveoli in distal generation 23 were increased proportionally to satisfy the required total inhalation flow rate. A similar behavior has been noticed where the volume redistributes from over-distended alveoli with reduced compliance to larger alveoli [26, 53].

### 2.3. Numerical Method

Following our previous, experimentally validated fluid-particle dynamics modeling [49], the alveolar fluid flow was simulated by solving the 3D Navier–Stokes equations on a moving mesh using an unsteady incompressible laminar flow solver with a user-enhanced commercial CF-PD package (CFX, Version 15 from ANSYS, Inc.; Canonsburg, PA). The particle transport was modeled using a Lagrangian approach assuming a dilute particle suspension. The major point forces acting on each particle are the drag (with Schiller–Naumann drag coefficient) and gravity [54]. The regional deposition of micron particles in human airways can be quantified in terms of the deposition fraction (DF):

(6) $$\begin{array}{c} \text{DF}=\frac{\text{number of deposited particles in a specific region}}{\text{number of particles entering the generation }16\text{ inlet}}. \end{array}$$

In order to reduce the computational effort and resources required for simulating the full alveolar model, each individual TBU/DBU was simulated independently. At the inlet of these TBUs/DBUs, an opening, i.e., entrainment, boundary condition was applied. The opening boundary condition, applying constant pressure, will allow flow in both directions. It will ensure that the physiological breathing mechanism (air being sucked in during inspirations and air being pushed out during expiration) is enforced. A wall deformation vector (depending on the local TBU/DBU) was applied on the alveoli surface and the rest of the airway wall was assumed to be rigid. At the outlet of the TBUs, a mass flow rate boundary condition was enforced in such a way that the outlet flow rate *Q*_D_ and flow rate due to the alveolar-wall motion *Q*_A_ together result in the inlet flow rate *Q*_G_ (i.e., flow rate per generation). The no-slip boundary condition was invoked at the wall boundaries (duct and alveoli) so that the fluid velocity matches with the wall velocity at the fluid wall interface. User-defined CFX Expression Language (CEL) functions were used to apply the wall deformation vector. In order to study the effects of inhalation and exhalation conditions on alveolar flow pattern, transient breathing profile with constant oral inhalation flow rates were assumed. Simulations were conducted for rest (15 LPM) breathing conditions with inhalation periods of 2 s and 4 s, corresponding to tidal volumes (TVs) of 500 ml and 1000 ml (Figure 1(f)). A simple breathing waveform was selected in order to compare the model predictions with experimental data sets in which subjects inhaled and exhaled at quasiconstant airflow rates. Clearly, *by controlling the alveolar-wall displacement, any inhalation waveform can be simulated, which implies that the model is capable of simulating actual breathing patterns.*

The geometrical models were meshed using ICEM from ANSYS, Inc. (Canonsburg, PA). The fluid domain of the single-generation alveolated models and the TBUs were discretized using tetrahedral elements with three prism layers. Prism layers were added to accurately capture the near wall gradients, while the DBU fluid domain consisted of only tetrahedral elements. A mesh convergence study was performed to ensure grid independent results by comparing the velocities at different cross sections as well as regional particle deposition values. Increasing the number of elements by a factor of 1.5 showed less than 1% of differences in these results. The final mesh for the single-generation, single-alveolated and multialveolated models had 100k and 280k elements while the TBUs had 2 million elements. In order to avoid large elemental deformation leading to negative volume, a coarser mesh was used for the 23^rd^ generation. The final mesh for the DBU had 400k elements.

Particles were distributed uniformly at the inlet with an initial velocity being the same as the inlet-air velocity. The outlet measurements (i.e., particle distributions) from the upper TBU outlets were exported at each time step to become the inlet condition for the next generation TBU, and the process was continued for all TBUs/DBU attached later on. During the exhalation phase, particle tracking starts from the suspended position at the end of inspiration, while the particles exiting the TBUs or DBUs were randomly injected into one of the outlets of the upper TBU, i.e., the outlets of the parent unit are considered as a single-injection plane from which the exited particles are reinjected. 20,000 particles per second were injected at the inlet of TBU 16–18. Particles were assumed to deposit on the walls on contact. Increasing the number of particles by a factor of 2 showed less than a 2% difference in the total acinar deposition results.

## 3. Results and Discussion

### 3.1. Alveolar Flow Pattern

**Figure 2:**
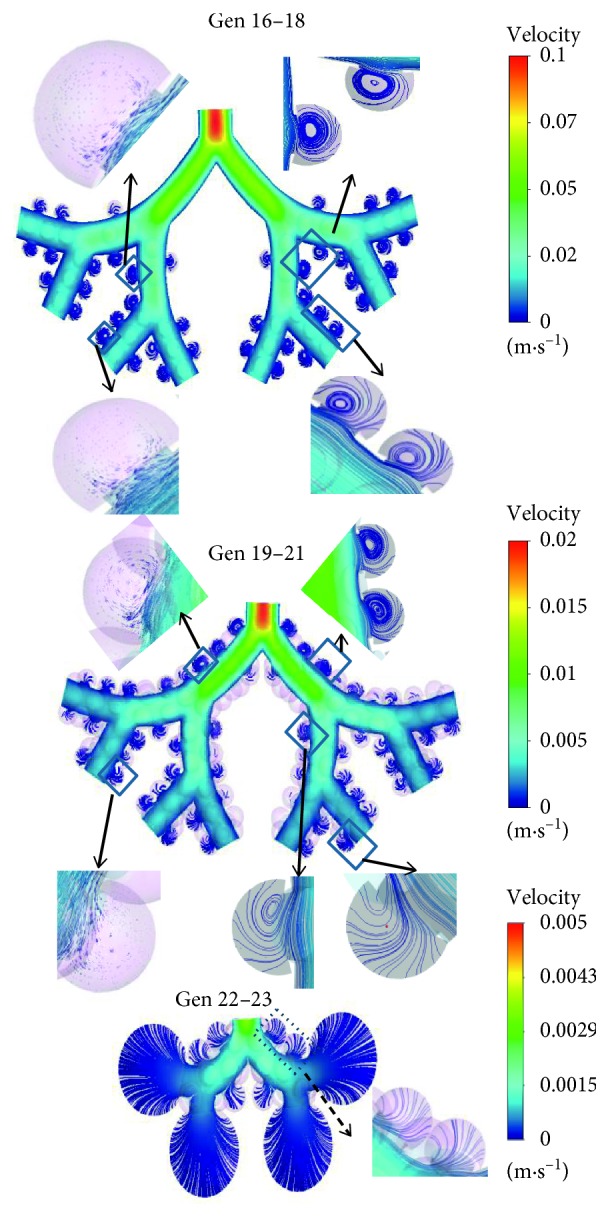
Enlarged view of the flow streamlines and velocity vectors inside alveolar airways [48].

The airflow through an alveolated duct depends on the rhythmic expansion and contraction rate of the alveoli. The flow patterns through different generations of the TBUs 16–18, TBUs 19–21, and DBUs 22-23 during inhalation with an inhalation flow rate of 15 LPM are shown in Figure 2. Enlarged views of the flow streamlines and velocity vectors inside alveolus are also displayed. The results indicate that the air in the alveolus of the proximal alveolar airways is strongly recirculating with substantially lower velocities when compared to the duct flow. Thus, only a very small fraction of the duct flow enters the alveoli; however, the shear flow across the alveolar mouth generates the recirculation region in the alveolar cavity. The presence of alveoli around the duct in conjunction with the alveolar pulling force generated from the alveolar-wall motion produces a deflection of a small amount of duct flow into the alveolar cavity. In the proximal alveolar airways, the size of the alveolar cavity is much less compared to the duct diameter. Hence, the amount of duct air entering the alveoli is also lower. However, within the TBUs, variations in recirculating flow intensity were noticed. A shown in Figure 2, the velocity vectors inside the alveolus in generation 17 shows rotating flow while the flow inside the alveolus of generation 18 exhibits a mixed radial-recirculating flow field. This is due to the decreased shear flow through the ducts. As the generation number increases, the flow is divided by half which in turn reduces the shear flow through the duct and hence the recirculating alveolar flow, thereby increasing the radial flow into the alveolus. The flow characteristics in TBUs 19–21 indicate that the intensity of the recirculating flow inside the alveolus is decreasing, while the radial flow is increasing. As a result, the flow inside an alveolus in generation 21 is mostly radial. Hence, it is expected that the mass transfer from the duct to the alveoli will increase in the distal lung generations. In DBUs 22-23, the flow pattern inside the alveolus is fully radial. The expansion of the alveolar sac model representing generation 23 generates a larger proportion of the inhalation flow rate into the lung so that the flow into the alveolar sac is fully radial. The Reynolds number in these distal lung regions under the assumed inhalation flow rate of 15 LPM is very low; hence, it can be assumed that the flow is quasisteady. The flow characteristics during exhalation were almost identical to the inhalation phase, other than that the direction of the flow was reversed. At the distal generations, the radial flow was directed from the alveolar cavity into the duct. Similarly, the direction of the recirculating flow in the proximal alveolar cavities was reversed with the change in phase. During the flow reversal from inhalation to exhalation, unsteady flow characteristics were not evident which may be due to the low inhalation flow rate.

### 3.2. Particle Transport and Deposition

**Figure 3:**
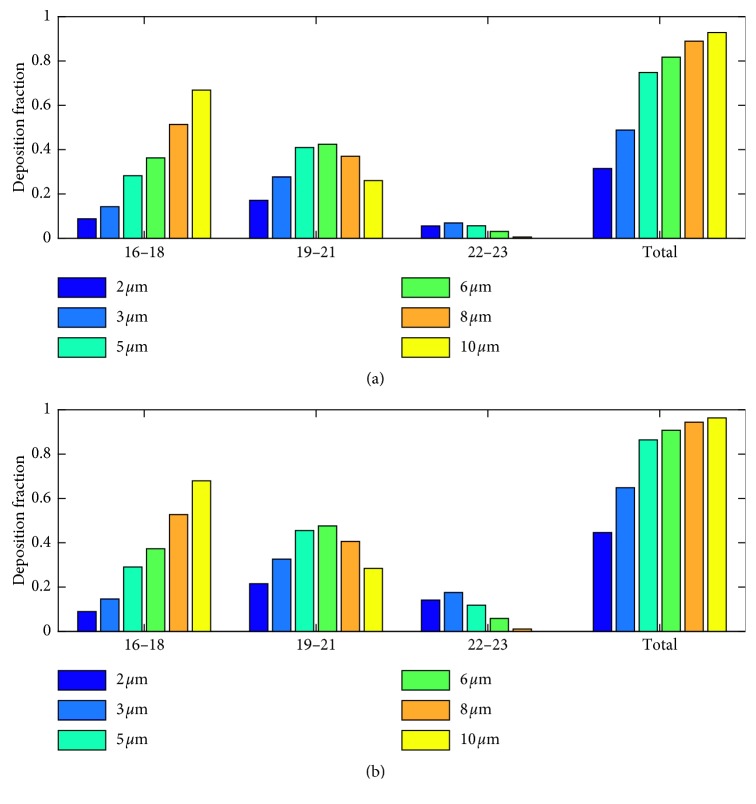
Particle deposition results for (a) TV 500 ml and (b) TV 1000 ml during inhalation phase.

Trajectories of particles with diameters 2 *μ*m to 10 *μ*m were simulated using the full acinar model. Gravity was assumed to act in the negative *z*-direction (Figure 1). Particle deposition results during inhalation phase are shown in Figure 3. The particle deposition was significantly influenced by particle size and tidal volume. The deposition fraction increased with the particle size and the tidal volume. There is an exponential increase in deposition for the lower-micron-size particles and as the particle size increased above 5 *μ*m, the increase in deposition reduces. Except for 2 *μ*m, more than 50% of the particles deposited during the inhalation phase itself. Even though total deposition trend showed an increase with particle size, segmental deposition varied over airway generations. Particle deposition increased with particle size in the proximal alveolar region (generation 16 to 18), increased with lower micron particle size, and decreased with upper micron particle size in the midalveolar region, but decreased with particle size in the distal alveolar region. The results also show that with the increase in tidal volume, more particles are being pulled into the distal lung airways, and hence the deposition in these distal lung generations increases. The number of particles deposited is higher in the midalveolar airways compared to other airway generations. The deposition in the proximal alveolar region (generation 16 to 18) is constant, for all the inhalation conditions considered. The results indicate that the increase of particle deposition with an increase in inhalation tidal volume is mostly due to the increased deposition in the distal alveolar airways.

**Figure 4:**
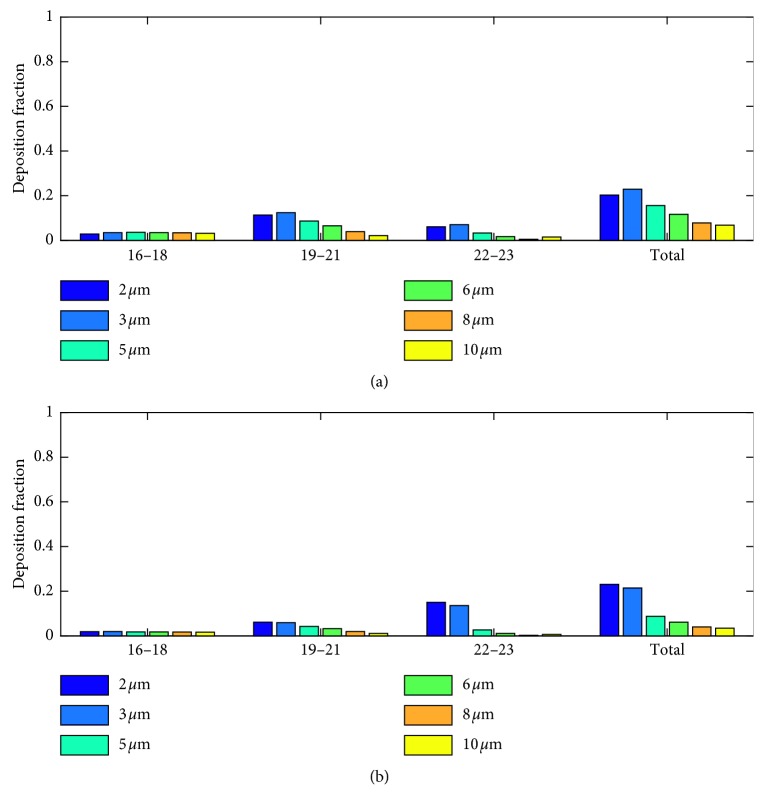
Particle deposition results for (a) TV 500 ml and (b) TV 1000 ml during exhalation phase.

The particle deposition results during exhalation phase are shown in Figure 4. The deposition fraction decreased with the particle size and did not show much variation with the tidal volume. Contrary to the inhalation phase, during exhalation phase particle deposition is higher in the midalveolar airways and in the distal alveolar airways. Lower-micron-sized particles have higher deposition in the mid- and distal airways, while larger micron particles tend to deposit in the proximal airways themselves. This can be attributed to the higher number of suspended larger micron particles in the proximal airways. These suspended particles are mostly particles that are being inhaled during the final inhalation phase. Due to the low inhalation flow rate, they reach the midalveolar airways so that during exhalation, these particles tend to deposit in the proximal alveolar airways due to sedimentation. Similar to the inhalation phase, the number of lower micron particles reaching the distal airways increased with tidal volume and hence the deposition in these airways.

**Figure 5:**
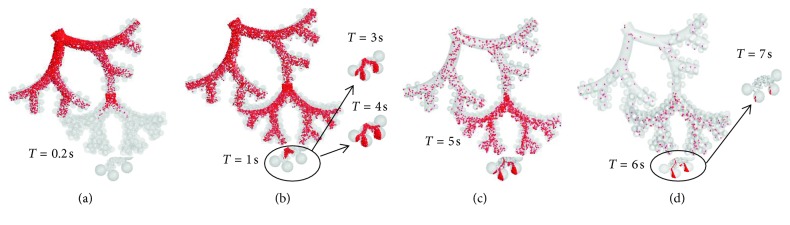
Position of the 3 *μ*m particles at selected time levels during an inhalation and exhalation phase with flow rate 15 LPM and tidal volume 1000 ml.

The positions of the 3 *μ*m particles at selected time levels during inhalation and exhalation phase with flow rate 15 LPM and tidal volume 1000 ml are shown in Figure 5. The particles were continuously injected at the inlet during the inhalation phase, and the particles were tracked until they were deposited or exhaled. Majority of the particles were convected with the duct flow during their transport through the lower lung generations. The particle front reached the second-level TBU within 0.2 s; however, due to the reduced flow velocity, the particle front penetrated into the DBU after 1 s. The transport and deposition of the particles were significantly influenced by the gravitational force. With the increase in generation number, more particles were found to enter the alveolar cavities which are oriented in the direction of the gravitational vector. Over time, a small number of particles were found to enter the alveoli in the proximal generations. These particles were mostly injected close to the lower airway surface which increased their chance to enter the alveoli. It is evident from the figure that with the increase in inhalation tidal volume, more particles tend to reach the distal alveolar generations. The 3 *μ*m particle-front reached generation 19 at around 0.15 s, while the 5 *μ*m particle-front took less time to reach the same generation. This is due to the higher gravitational pull. During the exhalation phase, the particles were pulled back into the upper lung generations. Particles present inside the alveolus were pulled out due to the contracting motion of the alveolus. However, the trajectories of the particles during exhalation were different when compared to inhalation, due to the effect of gravity. The gravitational pull during the exhalation phase has resulted in increased particle residence times of the particles and hence a higher deposition fraction.

### 3.3. Total Acinar Deposition and Comparison with In Vivo Measurements

**Figure 6:**
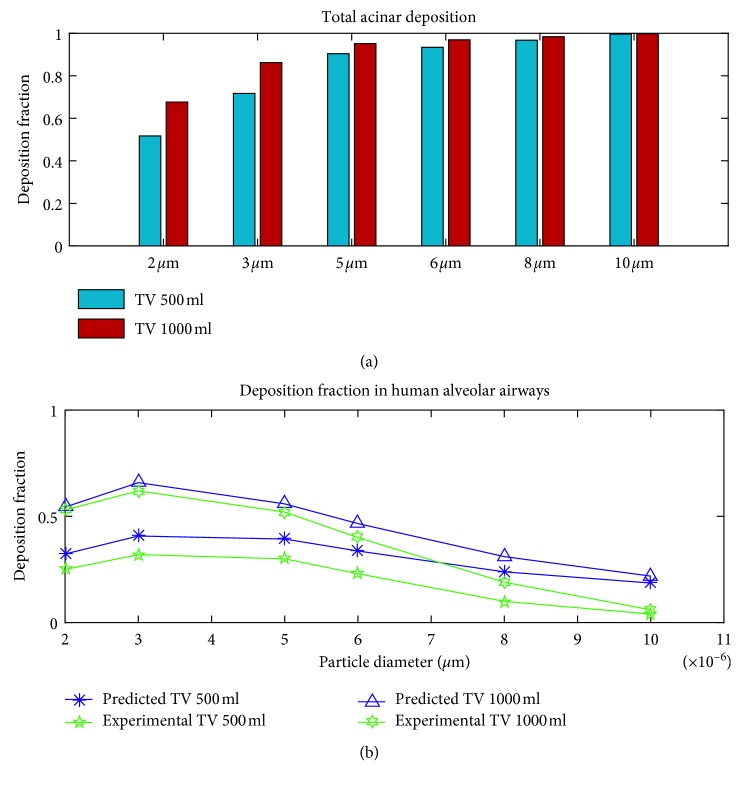
(a) Total acinar particle deposition results during a full breathing cycle (b) Comparison with in vivo measurements [55].

The total particle deposition results during a full breathing cycle are plotted in Figure 6(a). The number of particles remaining in suspension at the end of the first inhalation cycle was 3.75% of the total particles injected for 2 *μ*m and was less than 0.05% for other particle sizes. It is evident from the results that an increase in tidal volume and/or an increase in particle diameter will result in higher deposition in the acinar model.

Considering the deposition in the extra-thoracic and tracheobronchial region, particle deposition fraction in the alveolar region can be approximated from the model predicted deposition using the relation DF=*ϕ*(1 − *η*_*E*_)(1 − *η*_*F*_)*η*_*A*_. Based on these reported in vivo measurements [55] for deposition fraction in the extra-thoracic region (*η*_*E*_), tracheobronchial region (*η*_*F*_) (mean experimental deposition values for extra-thoracic region (*η*_*E*_), tracheobronchial region (*η*_*F*_) are reported in [55]), the volume fraction of the air coming to rest in the alveolar region (*ϕ*), and the present model predicted deposition *η*_*A*_, we translated the model predicted deposition *η*_*A*_, in the alveolar region into DF. This DF values were compared with the experimental measurements of alveolar deposition (Figure 6(b)) [55, 56]. The model predicted deposition results follow a similar trend and are in agreement with the in vivo alveolar deposition measurements [55]. Slight overprediction of alveolar deposition in the upper micron particle size range (around 10 *μ*m) could be either because of the underestimation of in vivo particle deposition in the upper airways. Other in vivo measurements have indicated higher deposition and large subject variability for upper micron particle around 10 *μ*m in the extra-thoracic and tracheobronchial deposition [56]. Considering that significant intersubject variability exists between measured human alveolar deposition data [56], the present model provides good approximation of deposition in human acinar region.

**Figure 7:**
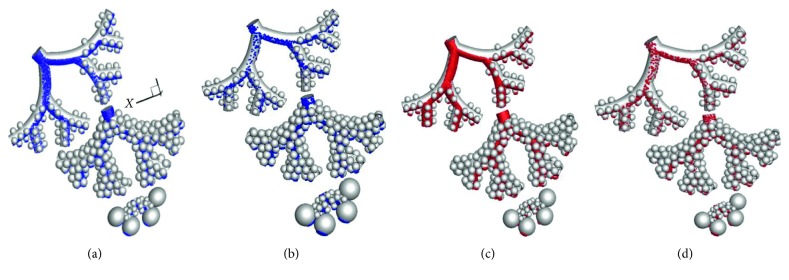
Location of the deposited particles (3 *μ*m and 5 *μ*m) in the full alveolar model during inhalation and exhalation phases with a tidal volume of 1000 ml. (a) 3 *μ*m inhalation. (b) 3 *μ*m exhalation. (c) 5 *μ*m inhalation. (d) 5 *μ*m exhalation.

The location of the deposited particles in the comprehensive alveolar model during inhalation with a tidal volume of 1000 ml is shown in Figure 7. The results indicate that the majority of the particles are deposited in the direction of the gravitational vector and the deposition is localized as the particles have predominantly entered into the alveolus with an opening in the direction of the gravitational vector. The deposition trends for the 3 *μ*m and 5 *μ*m particles are similar.

As mentioned, the full acinar model developed in this study is an anatomically inspired human alveolar model with eight generations of lung airways represented, using alveolated bifurcating tubular models, mimicking a full human acinar region. Among these, the first three generations are partially alveolated lung airways, and the rest are fully alveolated. In the present acinus model, the number of alveoli per triple bifurcation unit (TBU) is 84, 182, 25, and 4 in 16–18, 19–21, 22, and 23, respectively. One TBU was modeled per level with a mass flow rate boundary condition at the unresolved outlets. Specifically, in the acinar model there is one TBU representing generations 16–18, eight 19–21 TBUs, and sixty-four 22-23 DBUs. Hence, the total number of alveoli in this acinus model is 3396. In a symmetric lung model, there will be 2^15^ similar acini, which would result in more than 111 million alveoli per lung in the present model. If the 17 individual alveoli in the 23^rd^ generation, which was represented as a single spheroid is considered, there will be 7492 alveoli in total in the acinus model. For a symmetric lung model, there will be more than 245 million alveoli per lung. These digits are in close range with the recent estimations of the total number of alveoli in a human lung [1]. Recent numerical studies using multigenerational alveolar models [41] have indicated that a minimum number of fully alveolated airways (usually three) can predict realistic particle deposition results, i.e., adding additional generations had negligible impact on total acinar deposition. Therefore, the current model is reasonably accurate in representing a full acinar model and at the same time is computationally efficient. In this study, we used the ratio of alveolar flow to the airflow at the oral inlet, which is equal to the fractional alveolar volume in each generation. This approach makes it easy to incorporate the current acinar model into human whole-lung airway models, to simulate/predict the fate of inhaled toxic or therapeutic aerosols [48].

Previous multigenerational alveolar models have assumed homogeneous alveolar airway deformation without considering the variation in deformation rate with generation and in the number of alveoli per generation [19, 35, 41]. Model results from the present study have shown that the alveolar flow varies with airway generation and inhalation flow rate. The position of the alveolus determines the alveolus-wall deformation rate and the amount of convective mixing. The alveolar deformation rate in the current model depends on the mass flow rate through the TBUs and the alveolar volume fraction per TBU. Hence, the current model captures the variation in alveolar flow characteristics with airway generation number with respect to the number of alveoli per generation.

The particle deposition locations highlight the predominant deposition in the alveolar airway bifurcations. Although sedimentation is the dominant mechanism influencing particle deposition in the acinar region, the deposition trend indicates hotspots of particle deposition around the bifurcation ridges in the direction of gravitational orientation. While the gravitational orientation in the present study is fixed, recent investigations [41] showed that the total acinar deposition is independent of gravitational orientation; however, deposition hotspots were influenced by it [35].

The model results shows that light micron particles around 2 *μ*m are not completely expelled during one inhalation cycle and after each inhalation cycle, the suspended particles may reach deeper axial locations, while heavier particles (≥3 *μ*m) tend to deposit or exit within one inhalation cycle. The number of particles remaining suspended in the airway model at the end of the first inhalation cycle was around 3.75% of the total particles injected for 2 *μ*m and was less than 0.05% for other particle sizes. As expected, the number of particles that remain suspended in the domain reduced with increase in particle size and inhalation tidal volume [19, 35]. This indicates that once the smaller-micron-sized particles reach the alveolar region, it may not be completely expelled out within one inhalation cycle and it may remain there for longer duration providing higher particle residence time. These results are valuable insights for targeted delivery of therapeutic aerosols to distal lung regions.

## 4. Conclusions

A comprehensive, physiologically inspired acinar model has been developed which can efficiently simulate the physiological breathing mechanism and can predict variations in local alveolar flow pattern and particle deposition. The simulation results show that the alveolar flow pattern depends on the location of the alveoli. Dominant recirculating flow structures were noticed inside proximal lung generation alveoli, while intensity of the recirculating flow decreases while the radial flow increases in the midalveolar lung generations. In the distal alveoli, the airflow is completely radial. The particle simulation results indicate that microsphere deposition depends on the inhalation conditions and particle size. The particle deposition rate in the alveolar region increases with higher inhalation tidal volume and particle diameter. When the tidal volume is increased, more particles are pulled into the distal lung airways and hence the deposition in these distal lung generations increases. Irrespective of the tidal volume, the particle deposition fraction in the proximal alveolar region (i.e., generations 16 to 18) is constant, and the number of particles deposited is higher in the midalveolar airways compared to other airway generations. Contrary to the deposition results during the inhalation phase, particle deposition during exhalation is higher when the tidal volume is lower. This can be attributed to the large number of suspended particles depositing during the exhalation phase in the upper alveolar airways. The key mechanism for micron particle deposition in the alveolar lung airways is due to gravitational effects. However, the interplay between the breathing-induced wall motion and sedimentation significantly increases particle deposition.

## 5. Model Limitation and Future Work

The primary objectives were to develop a predictive modeling framework and provide computer simulation results for air-particle flow in a physiologically inspired whole acinar model. Presently, only one level of TBU is in-plane, i.e., it is rotated when connecting the next level. However, it has been noticed that the orientation of gravitational vector influences the deposition hot spots; hence, more detailed analyses may be required to study the effects of local airway orientation with respect to the gravitational vector on particle deposition. While relatively large nanoparticle transport and deposition can be accommodated with the present model, a separate analysis will focus on nanoparticles with *d*_p_ < 100 nm as part of the whole-lung airway simulation project. There are many uncertainties regarding the alveolar and duct biomechanics, e.g., recent studies have pointed out that the alveolar deformation is anisotropic [26]. Hence, further analyses are required considering neck and duct anisotropic deformations. Additionally, simulations with different inhalation conditions considering subject variability in breathing profiles will be carried out in future.

The limitations of this study also include the assumption of symmetric alveolar airways as well as the shape and size of the alveoli. Even though human lung-cast and tissue studies have indicated that there exist variations in the alveolar size and topology [11, 12], recent investigations have shown that these variations in alveolar topology might not significantly influence distal alveolar particle transport [30]; however, it may be significant in the upper alveolar airways [17, 20]. Indeed, with appropriate modifications such variations in alveolar topology and size could be incorporated into the current acinar model. The structural changes of the alveoli are still under debate. To reach a unifying hypothesis, further investigations are required. Additionally, we will build on the current modeling framework to study the influence of the lobar volumes on acinar deposition, using scaled models representing different lung lobes. A separate analysis will also focus on the effects of lung pathological conditions, such as emphysema, on alveolar airflow dynamics and particle deposition.

## Figures and Tables

**Table 1:** Geometric model parameters for a single alveolus.

		Literature comparison	Reference
Alveolus radius (AR)	0.105 mm	0.1–0.14 mm	Weibel [10]
			Harding and Robinson [13]
			Ma and Darquenne [35]
Neck radius (NR)	0.085 mm	0.085 mm	Harding and Robinson [13]
Alveolus depth (*D*)	0.2–0.22 mm	0.23 mm	Weibel [10]
			Harding and Robinson [13]
*D*/NR ratio	1.17–1.3	0.48–1.44	Harding and Robinson [13]

## Data Availability

The data used to support the findings of this study are available from the corresponding author upon request.
